# An algorithm to assess methodological quality of nutrition and mortality cross-sectional surveys: development and application to surveys conducted in Darfur, Sudan

**DOI:** 10.1186/1478-7954-9-57

**Published:** 2011-11-09

**Authors:** Claudine Prudhon, Xavier de Radiguès, Nancy Dale, Francesco Checchi

**Affiliations:** 1 Health and Nutrition Tracking Service, Geneva, Switzerland; 2 Department of International Health, University of Tampere, Finland; 3 Faculty of Infectious and Tropical Diseases, London School of Hygiene &Tropical Medicine, London, UK

## Abstract

**Background:**

Nutrition and mortality surveys are the main tools whereby evidence on the health status of populations affected by disasters and armed conflict is quantified and monitored over time. Several reviews have consistently revealed a lack of rigor in many surveys. We describe an algorithm for analyzing nutritional and mortality survey reports to identify a comprehensive range of errors that may result in sampling, response, or measurement biases and score quality. We apply the algorithm to surveys conducted in Darfur, Sudan.

**Methods:**

We developed an algorithm based on internationally agreed upon methods and best practices. Penalties are attributed for a list of errors, and an overall score is built from the summation of penalties accrued by the survey as a whole. To test the algorithm reproducibility, it was independently applied by three raters on 30 randomly selected survey reports. The algorithm was further applied to more than 100 surveys conducted in Darfur, Sudan.

**Results:**

The Intra Class Correlation coefficient was 0.79 for mortality surveys and 0.78 for nutrition surveys. The overall median quality score and range of about 100 surveys conducted in Darfur were 0.60 (0.12-0.93) and 0.675 (0.23-0.86) for mortality and nutrition surveys, respectively. They varied between the organizations conducting the surveys, with no major trend over time.

**Conclusion:**

Our study suggests that it is possible to systematically assess quality of surveys and reveals considerable problems with the quality of nutritional and particularly mortality surveys conducted in the Darfur crisis.

## Introduction

The prevalence of acute malnutrition and mortality rates are crucial indicators to benchmark the severity of a crisis, to track trends, and to inform funding and operational decisions [1,2]. Cross-sectional sample surveys are the main method currently used to estimate these indicators [3,4]. An adequate sampling design is indispensable to ensure the representativeness and accuracy of a survey. Moreover, standardized field data collection through suitable interview and measurement instruments and techniques is paramount to guarantee quality.

Despite recent improvements in standardization of nutrition and mortality survey methodology and analysis [5-7], errors in the field application of survey methods persist, potentially resulting in biased data and harmful operational decisions. Reviews of surveys carried out in various crisis settings have consistently revealed a lack of rigor in many nutritional [8-12] and most mortality surveys [10,11].

Cross-sectional surveys are generally conducted in relatively small areas corresponding to zones of intervention of non-governmental organizations (NGOs); therefore, an overall picture of the situation and its evolution at the regional and country level might be difficult to compose. Meta-analyses of mortality and nutrition data can help to infer crisis-wide trends [13]. Given the sparseness of data in most crises, it may be useful to include in the analyses survey reports with limited quality; however, to do this, weighting of surveys according to their relative quality seems necessary. More broadly, monitoring trends in the quality of surveys and the relative frequency of different errors can help to evaluate the strength of evidence on which many humanitarian decisions are predicated and fulfill a didactic role in improving field capacity for data collection.

The quality of a survey is determined by five components; coverage, sampling, nonresponse, measurements, and data processing. Errors could occur with each component. There are several guidelines for conducting and reporting on surveys in normal conditions published by the United States Office of Management and Budget (OMB) and the American Association for Public Opinion Research (AAPOR).

In this article, we describe an algorithm based on systematic and comparable criteria for analyzing nutritional and mortality survey reports to identify a comprehensive range of errors that may result in sampling, response, or measurement biases and score the quality of a survey as a whole. We apply the algorithm to surveys conducted in Darfur, Sudan between 2004 and 2008.

## Methodology

### Components of the algorithm

The algorithm is based on internationally agreed upon methods and best practices [2,14]. It is divided into seven components of quality checking: 1) selection bias of sampling design; 2) precision of cluster sampling (if done); 3) measurement biases for mortality; 4) measurement errors for nutrition; 5) response biases for mortality; 6) analysis errors for mortality; 7) analysis errors for nutrition [[15]; see Additional File 1]. These seven components are further grouped into three main sections: sample selection, data collection, and data analysis. For each of these, penalties are attributed for a list of possible more or less severe errors, and an overall score is built from the summation of penalties accrued by the survey as a whole. The criteria used are a mix in equivalent proportions of genuine errors and of lack of evidence of best practice.

### Penalties

A Delphi survey was conducted among experts to help determine consensus penalties for each criterion [16]. Experts commented on the algorithm and ranked each criterion from 0, defining a minor problem likely to have a negligible effect on the validity of the survey, to 5, a critical flaw indicating a serious error or an insufficient description that would thoroughly invalidate findings. Based on expert recommendations, the algorithm was refined further. The median penalty attributed by the experts was calculated for each error, and a geometric progression (a_n _= a * r^n-1^, with a = 1, r = 2.34, and n = 5) was used to rescale penalties to a range from 2 points, corresponding to 1 in the Delphi survey, to 70 points, corresponding to 5.

For nutritional surveys, selection of the sample, data collection, and analysis represented 37%, 31%, and 32% of the maximum possible penalty, respectively, when cluster sampling was used; 25%, 37%, and 38% when systematic random sampling was used; and 20%, 39%, and 41% when simple random sampling was used. For mortality surveys, which are arguably more vulnerable to nonsampling biases, these proportions were 48%, 38%, and 14% when cluster sampling was used; 36%, 46%, and 18% when systematic random sampling was used; and 31%, 50%, and 19% when simple random sampling was used.

### Overall quality scores

A continuous score, ranging from 0 to 1, is calculated from the sum of the penalties attributed to the survey, as follows:

$$\text{S}=1-\left(\text{L}∕\text{T}\right)$$

where S is the score, T the maximum number of penalty points a survey can accrue, and L the actual number of penalty points. For example, a score of 0.85 means that only 15% of the maximum penalty points were accrued by this survey. In a combined nutritional-mortality survey, the same error can have a different weight in the nutrition score compared to the mortality score; scoring for nutrition and mortality should therefore be interpreted independently.

T was calculated separately for different types of sampling design (simple random, systematic random, and cluster sampling), since each carries specific potential errors.

Based on the algorithm, a software application was developed for data entry of quality reviews and automatic calculation of scores [17].

### Testing of reproducibility

Reports of nutrition and mortality surveys conducted by NGOs and UN agencies in Darfur, Sudan between 2004 and 2008, previously gathered for the purpose of investigating trends in acute malnutrition and mortality [18], were used to test the algorithm's reproducibility and to apply it to a real-life scenario. Reports of rapid assessments that used convenience (i.e., nonrandom) sampling or did not measure the weight-for-height index for acute malnutrition were excluded from this collection of surveys.

To test the reproducibility of the algorithm, 30 survey reports from Darfur were randomly selected and three expert raters, blinded to one another's ratings, were asked to apply the algorithm to these surveys. Total penalties for mortality and nutrition surveys were calculated for each rater and the intraclass correlation coefficient was calculated to assess the consistency of rating [19]. Raters also provided their feedback on the use of the algorithm.

Each criterion for which the classification was different among the raters for more than 20% of the 30 survey reports analyzed was reviewed. Some of these criteria were reformulated to facilitate straightforward interpretation. A criterion about data cleaning procedure was deleted as it seemed impossible to judge with objectivity.

### Real-life application

The algorithm was further applied to the entire dataset of surveys from Darfur by one of the raters. Altogether, this dataset includes all nutrition and mortality surveys publicly released from the beginning of the crisis in 2004 to the end in 2008, and was chosen as it provides a case study of trends in quality across an entire crisis over time. Accordingly, the median and range of quality scores were calculated separately for mortality and nutrition surveys, overall, by year and by agency. Median scores between years and agencies were compared using a Kruskal-Wallis test [19]. The main reasons for critical flaws, main sources of penalties, and relative share of penalties by type of error were also computed. Some reports included more than one survey result if several areas or populations had been independently surveyed or if the survey was stratified. In this case, each survey result was considered as a separate survey, even if the methodology described in the report applied equally to the different survey strata. Calculation and statistical analyses were performed with Microsoft Excel and STATA (Stata Corporation, College Station, TX, USA).

## Results

One hundred and seventeen survey reports conducted by 19 NGOs and international agencies in Darfur, Sudan between June 2004 and December 2008 were collected. Twenty-eight preliminary reports were discarded from the analysis because the methodology was not reported with sufficient detail. In total, 108 mortality surveys and 100 nutrition surveys were analyzed. Of these, 107 mortality surveys and 97 nutrition surveys used cluster sampling, while one mortality and three nutrition surveys were conducted using systematic sampling.

### Reproducibility of the algorithm

The algorithm was independently applied by three raters on 30 randomly selected survey reports from 11 NGOs, including 29 mortality surveys and 30 nutrition surveys. Of the 108 quality criteria in the algorithm, raters disagreed on whether the criterion was fulfilled in more than 50% of the surveys for seven (6.5%) criteria and in 20% to 50% of the surveys for 20 (18.5%) criteria. The Intra Class Correlation coefficient was 0.79 for mortality surveys and 0.78 for nutrition surveys. The three-way average Intra Class Correlation coefficient (the three raters considered as a group of raters rather than as independent raters) was 0.92 and 0.91 for mortality and nutrition surveys, respectively.

### Quality of Darfur surveys

Twenty-six mortality survey results (24%) and 13 nutrition survey results (13%) featured a critical flaw. Critical flaws were due to unclear description of the first stage sampling of clusters for six mortality surveys and three nutrition surveys, unclear description of household sampling in the last stage of cluster sampling for 14 mortality surveys and four nutrition surveys, and to both of these criteria for six mortality and six nutrition surveys. For some of these surveys, the methodology used was not described at all (eight mortality surveys and nine nutrition surveys). For the others, a reference to a guideline or paper was given (18 mortality surveys and three nutrition surveys) but pointed to the description of several choices of first or second step cluster sampling, making it impossible for the reader to identify which methodology was used. Most of the surveys with critical flaws were conducted in 2004 (10 mortality and eight nutrition surveys) and 2005 (13 mortality and two nutrition surveys).

**Figure 1:**
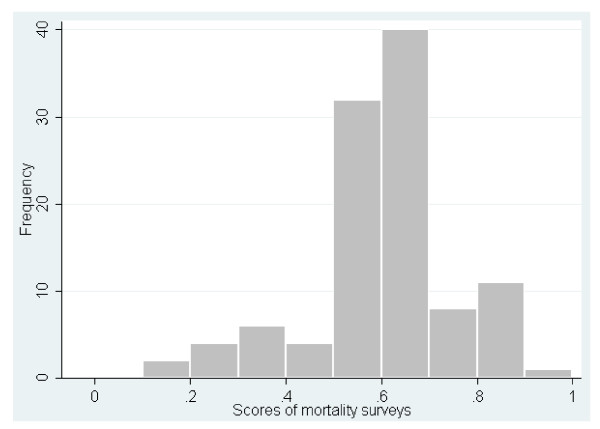
**Frequency distribution of quality scores for 108 mortality surveys, Darfur, Sudan, June 2004 - December 2008**.

**Figure 2:**
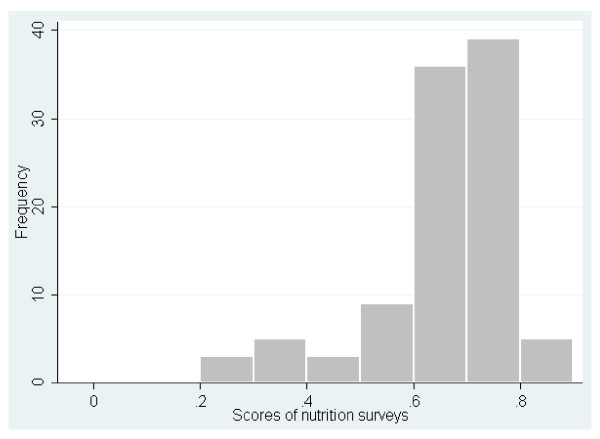
**Frequency distribution of quality scores for 100 nutrition surveys, Darfur, Sudan, June 2004 - December 2008**.

When surveys with critical flaws were included in the analysis, the overall median score was 0.60 (ranging from 0.12 to 0.93) for mortality surveys (Figure 1) and 0.69 (ranging from 0.23 to 0.86) for nutrition surveys (Figure 2). When surveys with critical flaws were excluded from the analysis, the scores for mortality surveys varied between 0.42 and 0.93, with the majority falling between 0.50 and 0.70, while the scores for nutrition surveys varied between 0.50 and 0.86, with the majority between 0.60 and 0.80. The overall median of the scores for mortality and nutrition surveys was 0.61 and 0.71, respectively. The 15 highest-score mortality surveys (0.79-0.93) were mainly conducted by two organizations well known for their expertise in epidemiology. By contrast, nine organizations shared the 15 best scores for nutrition (0.76-0.86), including the above-mentioned two organizations.

Median scores for mortality and nutrition surveys, excluding surveys with critical flaws, varied among organizations from 0.54 to 0.80 and from 0.63 to 0.75, respectively (Table 1), and were significantly different for both mortality (Kruskal-Wallis Chi-square = 38.86, 13 degrees of freedom [DF], p = 0.0002) and nutrition (Kruskal-Wallis Chi-square = 38.34, 14 DF, p = 0.0005). Median scores among agencies were even more different when surveys with critical flaws were taken into account, varying from 0.38 to 0.80 for mortality surveys (Kruskal-Wallis Chi-square = 43.82, 15 DF, p = 0.0001) and from 0.38 to 0.75 for nutrition surveys (Kruskal-Wallis Chi-square = 44.93, 15 DF, p = 0.0001).

**Table 1:** Quality scoring of 108 mortality surveys and 100 nutrition surveys by organization (for organizations having conducted more than one survey), Darfur, Sudan, June 2004 - December 2008

Organizations	Total number of survey results Mortality (Nutrition)	Number of survey results with critical flaws Mortality (Nutrition)	Surveys without critical flaws		All surveys	
			
			Score mortality Median (range)	Score nutrition Median (range)	Score mortality Median (range)	Score nutrition Median (range)
A	35 (36)	1 (1)	0.60 (0.38-0.83)	0.70 (0.63-0.86)	0.60 (0.38-0.83)	0.70 (0.53-0.86)
B	13 (13)	0 (0)	0.80 (0.79-0.93)	0.75 (0.74-0.84)	0.80 (0.79-0.93)	0.75 (0.74-0.84)
C	6 (6)	0 (0)	0.57 (0.49-0.68)	0.65 (0.57-0.77)	0.57 (0.49-0.68)	0.65 (0.57-0.77)
D	7 (7)	0 (0)	0.60 (0.41-0.63)	0.64 (0.60-0.70)	0.60 (0.41-0.63)	0.64 (0.60-0.70)
E	4 (4)	0 (0)	0.54 (0.42-0.61)	0.64 (0.61-0.67)	0.54 (0.45-0.61)	0.65 (0.61-0.67)
F	2 (2)	0 (0)	0.66 (0.66-0.66)	0.69 (0.65-0.73)	0.66 (0.66-0.66)	0.69 (0.65-0.73)
G	3 (3)	1 (1)	0.66 (0.62-0.70)	0.77 (0.75-0.79)	0.62 (0.16-0.70)	0.75 (0.23-0.79)
H	13 (13)	7 (7)	0.64 (0.46-0.84)	0.66 (0.56-0.80)	0.38 (0.24-0.84)	0.38 (0.31-0.80)
I	5 (8)	2 (2)	0.62 (0.56-0.70)	0.66 (0.50-0.78)	0.57 (0.12-0.70)	0.61 (0.23-0.78)
J	0 (2)	0 (1)	-	-	-	0.58 (0.55-0.61)
K	14(0)	14 (0)	-	-	0.64 (0.51-0.64)	-

There was no apparent trend in median score for either mortality or nutrition surveys considering the year of survey completion, when surveys with critical flaws were discarded from the analysis (Table 2) (mortality: Kruskal-Wallis Chi-square = 0.24, 4 DF, p = 0.99; nutrition: Kruskal-Wallis Chi-square = 2.65, 4 DF, p = 0.62). Ranges were larger for mortality scores than for nutrition scores for all years. By contrast, when surveys with critical flaws were included in the analysis, difference in median scores among years was borderline significant (mortality: Kruskal-Wallis Chi-square = 9.38, 4 DF, p = 0.052; nutrition: Kruskal-Wallis Chi-square = 10.97, 4 DF, p = 0.027), especially because of low median scores in 2004.

**Table 2:** Quality scoring of 108 mortality surveys and 100 nutrition surveys by year, Darfur, Sudan, June 2004 - December 2008

Year	Number of organizations	Total number of survey results Mortality (Nutrition)	Number of survey results with critical flaws Mortality (Nutrition)	Surveys without critical flaws		All surveys	
				
				Score mortality Median (range)	Score nutrition Median (range)	Score mortality Median (range)	Score nutrition Median (range)
2004	9	17 (19)	10 (8)	0.59 (0.46-0.93)	0.67 (0.50-0.84)	0.51 (0.24-0.93)	0.55 (0.28-0.84)
2005	11	39 (29)	13 (2)	0.61 (0.49-0.80)	0.66 (0.56-0.79)	0.64 (0.16-0.80)	0.66 (0.23-0.79)
2006	7	21 (21)	1 (1)	0.61 (0.51-0.83)	0.73 (0.57-0.86)	0.61 (0.38-0.83)	0.73 (0.53-0.86)
2007	5	19 (19)	1 (1)	0.60 (0.42-0.82)	0.71 (0.63-0.77)	0.60 (0.12-0.82)	0.71 (0.23-0.77)
2008	5	12 (12)	1 (1)	0.61 (0.43-0.73)	0.70 (0.61-0.81)	0.61 (0.27-0.73)	0.70 (0.42-0.81)

Around 90% of the penalty points were due to lack of evidence from the survey reports, ranging between 70% to 99% in mortality surveys and between 49% to 100% in nutrition surveys. Of the three sections of the algorithm, "data collection" accrued the highest proportion of maximum penalty points for surveys (54% for mortality, 49% for nutrition), while around 30% of maximum penalty points were accrued for the section on "selection of basic sampling." The least problematic section was "analysis, " with only about 15% of maximum penalty points accrued.

For mortality, the most striking error in data collection procedures was the absence of a structured questionnaire with explicit questions and sequences of questions, which limits standardization and reproducibility of interviews among different households for the same interviewer and among interviewers (Table 3). Moreover, most of the time, only aggregated data were collected, precluding a detailed person-time analysis. For nutrition, the major problem of quality of data collection was the absence of detailed descriptions of the accuracy of the material used for height and mid-upper arm circumference (MUAC) measurements (Table 4). Reports also lacked clarity about on-site supervision and data checking for both mortality and nutrition. The most frequent flaw in the selection of the basic sampling unit for mortality and nutrition surveys was that the number of nonresponding households was not reported.

**Table 3:** Main sources of penalty for 82 mortality surveys, Darfur, Sudan, June 2004 - December 2008

	Mortality surveys penalized for the criterion (%)
**Selection of basic sampling unit**	
1) No cross-checking of the source for population size	50
2) Household selection in cluster sampling done by the "spin the pen" method	77
3) Further household selection by proximity with no interval between households	74
4) No definition of household	58
5) No description of revisit strategy	50
6) Number of nonresponding households not reported	98
**Mortality data collection**	
1) No evidence of the use of a structured questionnaire including explicit questions and sequences of questions	83
2) No evidence that questionnaire/tally sheet was prepiloted before data collection	55
3) If multipurpose survey, no evidence of mortality questions at the beginning of interview and before anthropometric measurements	100
4) Aggregated questionnaire/tally-sheet only	87
5) No use of a calendar with salient dates for estimating date of deaths	68
6) No evidence of field supervision, including direct observation of interviews by investigators	85
7) No evidence that data collection forms were checked at the end of each day by investigators	94
8) No evidence that the interviewers explained that the survey was not part of a registration effort and would not affect relief allocation for the household	87
**Analysis of mortality data**	
1) Standard errors or confidence intervals were not reported in the results	58
2) No evidence that adjustment of standard errors for clustering was done, when relevant	13

**Table 4:** Main sources of penalty for 87 nutrition surveys, Darfur, Sudan, June 2004 - December 2008

	Nutrition surveys penalized for the criterion (%)
**Selection of basic sampling unit**	
1) No cross-checking of the source for population size	51
2) Household selection in cluster sampling done by the "spin the pen" method	76
3) Further household selection by proximity with no interval between households	72
4) No definition of household	60
5) No description of revisit strategy	53
6) Number of nonresponding households not reported	92
**Nutrition data collection**	
1) No evidence that standardization of measurements was performed	69
2) No evidence that interviewers explained that the survey would not affect relief allocation for the household	87
3) No evidence of field supervision, including direct observation of interviews by investigators	85
4) No evidence that data collection forms were checked at the end of each day by investigators	95
5) Absence of detailed description of the accuracy of the material used for weight measurements	49
6) Absence of detailed description of the accuracy of the material used for height measurements	100
7) Absence of detailed description of the accuracy of the material used for MUAC measurements	97
8) Inclusion of children based on age or height	74
9) No report of the percentage of children with age estimated from birth certificate	48
**Analysis of nutrition data**	
1) No information on number of data discarded from the analysis because of child's inclusion errors or anthropometric flags	42
2) No evidence that adjustment of standard errors for clustering was done, when relevant	38
3) No information about standard deviation of weight-for-height index	70

## Discussion

### Use of the algorithm and its limitations

The analysis and interpretation of nutrition and mortality data in crisis settings are notoriously challenging tasks. First, the quality of data is often questioned. Second, the coverage of data is frequently sparse, limiting inference beyond a few sites or time periods for which estimates are available. The algorithm presented in this paper allows for standardization of the assessment of quality of nutrition and mortality surveys according to systematic and comparable criteria.

The algorithm is intended to be used by epidemiologists experienced in nutrition and mortality surveys but without prior training on the algorithm itself. The test of reproducibility that we conducted with three different raters suggested that the algorithm was sufficiently reliable to broadly benchmark a given survey's quality. The three-way average Intra Class Correlation also suggested that quite good reliability could be achieved by having two raters per survey. This could be applied to the most difficult surveys and the most critical and sensitive situations. Depending on the familiarity with the algorithm, rating of a survey takes between 30 minutes and one hour.

The algorithm can be used to gauge the overall quality of data at the regional or country level. It can also be utilized to assess trends in quality of surveys and the impact of initiatives aimed at improving survey practice. Furthermore, agencies can apply the algorithm to the surveys they perform in order to review their overall quality, investigate their strengths and weaknesses, and take adequate measures to improve major flaws. In this respect, the algorithm could serve as a training tool, but also to help enforce performance targets.

The main limitation of the algorithm is that it is based on information available in the reports, which for most of the criteria may partly reflect the completeness of the report. Because the algorithm generally assumes that a given best practice was not implemented if there is no mention of it in the report, it may be biased towards worst case scenarios, and the quality of some surveys might in fact be better than evaluated through the algorithm. However, in the absence of raw data, evaluating the quality of surveys from reports, including the missing information, is the best available proxy for survey quality. The writing of lengthy survey reports might appear cumbersome but is of paramount importance and is inherent to sound epidemiological practices to document procedures and allow others to assess the quality of data. This is part of the epidemiological rigor to thoroughly describe the methodology used in surveys and trials to allow others to evaluate the soundness of results. Data with established good validity could have a stronger influence on decision-makers and donors. Indeed, the criteria included in the algorithm can be used as a basis for minimum reporting.

As shown in the algorithm, some analysis errors (e.g., lack of weighting or design effect adjustment) can be rectified using raw data; however, these are seldom available. Re-analysis of datasets can also reveal potential quality problems. For example, the Emergency Nutrition Assessment (ENA) software performs a series of plausibility and quality checks on anthropometric indices, mainly based on the frequency of outliers and departures from normality, and based on these attributes an overall score to the survey [7]. It could be possible to combine the quality score of our algorithm (which focuses more on methods and procedures) and the quality score of ENA (which focuses on the data) into a summary score of a given nutritional survey.

The precision of survey results was not included in the scoring system because it was not seen as a quality issue but as an impediment to meaningful interpretation of survey results.

Lastly, our algorithm necessarily relies on arbitrary decisions on the relative gravity of different errors. We have attempted to address this limitation through a Delphi survey, a standard approach to eliciting expert opinions. As the algorithm is adopted for routine practice, penalties for individual errors could be revisited and re-adjusted if necessary.

### Quality of surveys in Darfur

Darfur has been one of the major humanitarian operations in the past few years [20]. Our study suggests considerable problems with quality of nutritional and particularly mortality surveys conducted in this crisis. In such a highly politicized environment, it is especially crucial that evidence is robust. Poor quality of surveys may have contributed to conflicting estimates of the number of deaths in Darfur in the early period of the crisis [21].

Previous reviews conducted in other humanitarian crises of the quality of nutrition and mortality surveys based on survey reports have shown that quality was generally limited [8-12]. One study, however, suggested that the quality of nutrition (but not mortality) surveys conducted between 1993 and 2004 in 17 countries improved over time [11]. Our study also suggests some improvements in quality of mortality and nutrition surveys between 2004 and 2008. However, this might be due to the fact that the two agencies that contributed to the majority of surveys with critical flaws only conducted surveys in 2004 and 2005. When surveys with critical flaws were removed from the analysis, no difference was evidenced.

The proportion of critical flaws appeared high, especially for mortality surveys. However, it is possible that some of the 16% of mortality surveys that, instead of clearly describing methodology, provided a reference for methodology of first and second stage cluster sampling, were actually conducted adequately. They had overall no more errors than surveys without critical flaws.

There were obvious differences in median score among agencies for both mortality and nutrition surveys. The same discrepancy was shown in a paper analyzing survey quality from 1993 to 2004 [11], suggesting that international initiatives have failed to improve uniformly survey quality across agencies.

The release of manuals on nutrition and mortality survey methodology [2,14] and the development of user-friendly tools to analyze nutrition and mortality surveys [7] have probably contributed to the standardization of survey methodology and improvement of data analysis. Our reading of multiple survey reports, however, suggested that these tools are sometimes used in a simplistic way with no understanding of the underlying epidemiology principles. Training initiatives on survey methodology have recently intensified and are the best way to improve survey quality. Training should not only include the "how" to conduct a survey but also the "why, " i.e., basic principles in epidemiology and statistics. This would increase survey investigators' understanding of the rules governing survey methodology and analysis of data. A better knowledge of epidemiology principles should also be promoted among policymakers to guide more rational commissioning of surveys and allocation of resources based on correct interpretation of findings.

## Conclusion

Our study suggests that it is possible to systematically assess quality of surveys and reveals considerable problems with the quality of nutritional and particularly mortality surveys conducted in the Darfur crisis. Improving survey quality will strengthen the evidence-based funding and operational decisions.

## Competing interests

The authors declare that they have no competing interests.

## Authors' contributions

All authors read and approved the final manuscript and agreed with the manuscript's results and conclusions. CP, XdR and FC designed the study. CP analyzed the data. CP, XdR, and ND participated in the collection of the data and did experiments for the study. CP wrote the first draft of the paper and all authors revised the manuscript.

## Supplementary Material

Additional file 1 **Additional file **1**shows the algorithm for checking the quality of mortality and nutrition surveys**. Click here for file

## References

[B1] The Sphere Project Humanitarian charter and minimum standards in disaster response 2004 Geneva: The Sphere Project. 10.1111/j.0361-3666.2004.00245.x 20958782

[B2] SMART Measuring mortality, nutritional status, and food security in crisis situations: SMART methodology, version 1 http://www.smartmethodology.org/images/stories/SMART_Methodology_08-07-2006.pdf Accessed December 1, 2010

[B3] Bostoen K Bilukha O Fenn B Methods for health surveys in difficult settings: charting progress, moving forward ETE 2007 4 13

[B4] Checchi F Roberts L Documenting Mortality in Crises: What Keeps Us from Doing Better? PLoS Med 2008 5 7 e146 10.1371/journal.pmed.0050146 18597552 PMC2443202

[B5] Salama P Spiegel P Talley L Lessons learned from complex emergencies over past decade Lancet 2004 364 1801 1813 10.1016/S0140-6736(04)17405-9 15541455

[B6] Young H Borrel A Holland D Public nutrition in complex emergencies Lancet 2004 364 1899 1909 10.1016/S0140-6736(04)17447-3 15555671

[B7] ENA; software for Emergency Nutrition Assessment http://www.nutrisurvey.net/ena/ena.html Accessed December 1, 2010

[B8] Boss LP Toole MJ Yip R Assessments of mortality, morbidity, and nutritional status in Somalia during the 1991-1992 famine JAMA 1994 272 371 376 10.1001/jama.272.5.371 8028168

[B9] Garfield R Studies on young child malnutrition in Iraq Nutr Rev 2000 58 269 277 11060997 10.1111/j.1753-4887.2000.tb01880.x

[B10] Grais FR Luquero FJ Grellety E Learning lessons from field surveys in humanitarian contexts: a case study of field surveys conducted in North Kivu, DRC 2006-2008 Conflict and Health 2009 3 8 10.1186/1752-1505-3-8 19744319 PMC2753557

[B11] Prudhon C Spiegel PB A review of methodology and analysis of nutrition and mortality surveys conducted in humanitarian emergencies from October 1993 to April 2004 ETE 2007 4 10 17543104 10.1186/1742-7622-4-10 PMC1906753

[B12] Spiegel PB Salama P Maloney S Quality of malnutrition assessment surveys conducted during famine in Ethiopia JAMA 2004 292 613 618 10.1001/jama.292.5.613 15292087

[B13] Degomme O Guha-Sapir D Patterns of mortality rates in Darfur conflict Lancet 2010 375 294 300 10.1016/S0140-6736(09)61967-X 20109956

[B14] World Food Programme/Center for Disease Control and Prevention A Manual: Measuring and Interpreting Malnutrition and Mortality 2005 Rome: WFP

[B15] Health and Nutrition Tracking Service Algorithm for quality checking of mortality and nutrition surveys http://www.thehnts.org/en/27 Accessed December 1, 2010

[B16] Hsu CC Sandford BA The Delphi Technique: Making Sense Of Consensus Practical Assessment, Research and Evaluation 2007 12 10

[B17] Health and Nutrition Tracking Service Surveys, a spreadsheet application for data entry and automatic analysis of nutrition and mortality surveys, and quality scoring http://www.thehnts.org/en/27 Accessed December 1, 2010

[B18] Nielsen J Trends in malnutrition and mortality in Darfur, Sudan, between 2004 and 2008: a meta-analysis of publicly available surveys. 2011 40 4 971 984 21296853 10.1093/ije/dyr010

[B19] Armitage P Berry G Statistical methods in medical research 1994 Third Oxford: Blackwell Science

[B20] Moszynski P Violence in Darfur region is jeopardizing world's largest aid operation BMJ 2006 333 319 16902208 10.1136/bmj.333.7563.319-a PMC1539085

[B21] GAO Report to congressional requesters. Darfur crisis- Death estimates demonstrate severity of crisis, but their accuracy and credibility could be enhanced 2006 http://www.gao.gov/new.items/d0724.pdf Accessed December 1, 2010

