# Supplementary material for: An algorithm to assess methodological quality of nutrition and mortality cross-sectional surveys: development and application to surveys conducted in Darfur, Sudan
Source: Popul Health Metr. 2011 Nov 9;9:57. doi: 10.1186/1478-7954-9-57 (PMC3231955; doi:10.1186/1478-7954-9-57)

H N T S · S I S N
Health and Nutrition Tracking Service
Service d'Information de Santé et Nutrition

Algorithm for quality checking of mortality and nutrition surveys

The objective of the quality checking is to assess the quality of nutrition and mortality surveys through the use of an algorithm, in order to make optimal use of data available when combining information from different sources but taking into account their relative plausibility.

The algorithm is made of objective criteria based on internationally agreed methodologies and best practices for analysing nutrition and mortality survey reports, thus allowing a relatively junior data analyst to carry out a reliable review. It breaks down the assessment of a given survey report into detailed components, including 1) Selection bias of sample; 2) Precision of cluster sampling; 3) Measurement biases for mortality; 4) Measurement errors for nutrition; 5) Response biases for mortality; 6) Reliability of analysis for mortality; and 7) Reliability of analysis for nutrition. While some criteria reflect the actual practice during the surveys, most were formulated to take into account the absence of information in the survey reports and therefore are a measure of the completeness of the survey reports, which, in absence of raw data, is the best available proxy for survey quality.

Penalties, ranking from 0: a minor problem which is likely to affect only slightly the validity of the survey to 5: a fatal error which will indicate that the survey methodology has a serious problem and that the survey should be discarded from the analysis, are applied to the individual criteria of quality. Penalties were attributed according to the results of a Delphi survey seeking agreement among HNTS' Expert Reference Group.

A continuous score is subsequently calculated from the sum of the penalties to provide an overall quality index that can be used as a weight in later analysis. The Score (S) is calculated as follow: $S = 1 - (L/T)$

With T = Total number of penalty points a theoretical survey could accrue if it had the maximum possible penalty for each component.

The score can take any value from 0 to 1. The higher the score, the better the survey, e.g. a score of 0.85 means that only 15% of the maximum penalty points were accrued by this survey.

Based on this algorithm, a software application was developed for quality data entry and automatic calculation of quality score. The scoring of reports can be completed with a reanalysis of raw data where available or with quality checks of raw data from ENA software for SMART.

The application allows 1. data entry of nutrition and mortality survey results and automatic analyses of trends; 2. quality data entry and automatic calculation of quality score.
The application is an Excel® workbook composed of 7 sheets:

- a [_geo] sheet for setting up the geographical information (i.e. administrative levels for a given country),
- a [Lists] sheet displaying a list of all the countries in the world and their corresponding ISO codes, and a list of the scoring variables and their corresponding penalty points,
- a [Data entry] sheet for opening the data entry form and the user guide,
- three sheets presenting the results of the automatic analyses,
- a [Data] sheet containing the dataset

Acknowledgments
This algorithm was originally developed by Francesco Checchi and adapted by Claudine Prudhon and Xavier de Radiguès, who has also developed the software application for the algorithm. We would like to acknowledge the members of the HNTS' Expert Reference Group and Steering Committee who provided comments and feed-back on the algorithm, as well as Nancy Dale, Bridget Fenn and Catherine Sprecher-Bachy who piloted the algorithm and software application.

References
ENA (2010) Software for emergency nutrition assessment.
http://www.nutrisurvey.net/ena_delta
SMART (2006) Measuring mortality, nutritional status, and food security in crisis situations: SMART methodology, version 1
http://www.smartmethodology.org/images/stories/SMART_Methodology_08-07-2006.pdf
Surveys (2010) Software for entering and analysing survey data and quality. HNTS.
http://www.thehnts.org
World Food Program & Centre for Disease Control (2005). A Manual: Measuring and Interpreting Malnutrition and Mortality. Rome: WFP.
www.unscn.org/en/resource_portal
World Health Organization (1983) Measuring change in nutritional status.
http://whqlibdoc.who.int/publications/1983/9241541660.pdf

LEGEND

··········▶	**True (And)**
──────▶	**False (Or)**
4	**Penalty attributed, from 0: minor problem which is likely to affect only slightly the validity of the survey to 5: fatal error which will indicate that the survey methodology has a serious problem and that survey should be discarded from the analysis**
**No or unclear Description**	**Information missing from the report, qualify completeness of reports**
(dashed box)	**Can be rectified through reanalysis if population data or other proxies of Primary Sampling Unit size (e.g. satellite data) are available**
(dotted box)	**Can be rectified through reanalysis of dataset or conservative assumption about Intra Class Correlation and Design Effect**

1. SAMPLING - SELECTION BIAS

1.1 Sampling universe and sampling frame

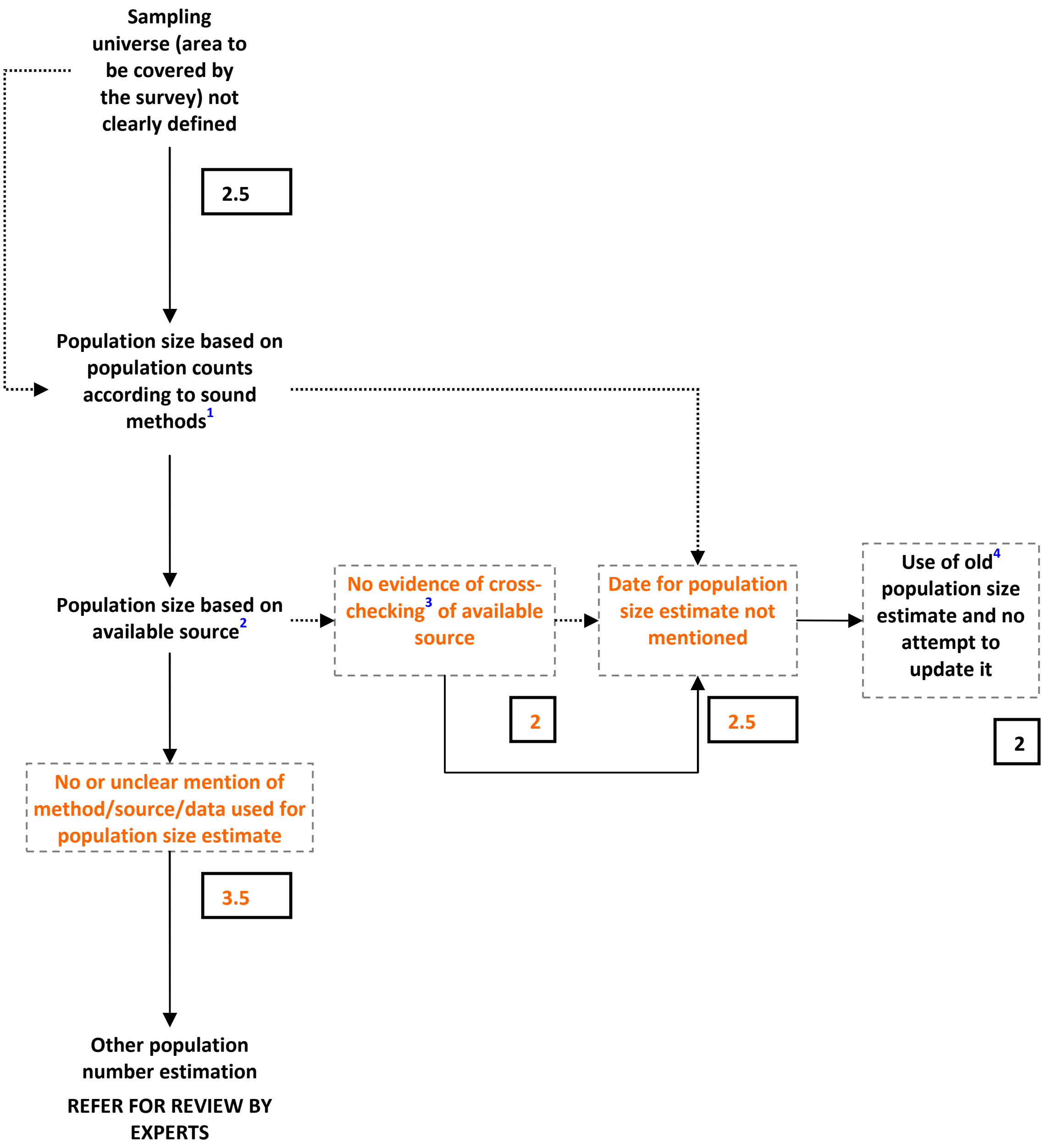

[1] Sound methodologies include census, registration, count of residential structure with survey of structure occupancy and areas sampling such as quadrat and T-square.

[2] If population size is updated using an arbitrary population increase rate equally applied to all geographical areas, although not good practice, this is not to be penalised as it does not have major implications for PPS.

[3] It is preferable that the source used for the survey is cross-checked with other data on population size.

[4] Indicative benchmark, 3 months for unstable population, 3 years for stable population.

1. SAMPLING - SELECTION BIAS (Continued)

1.2 Sampling design within explicit strata (or entire sampling universe if no explicit stratification done)

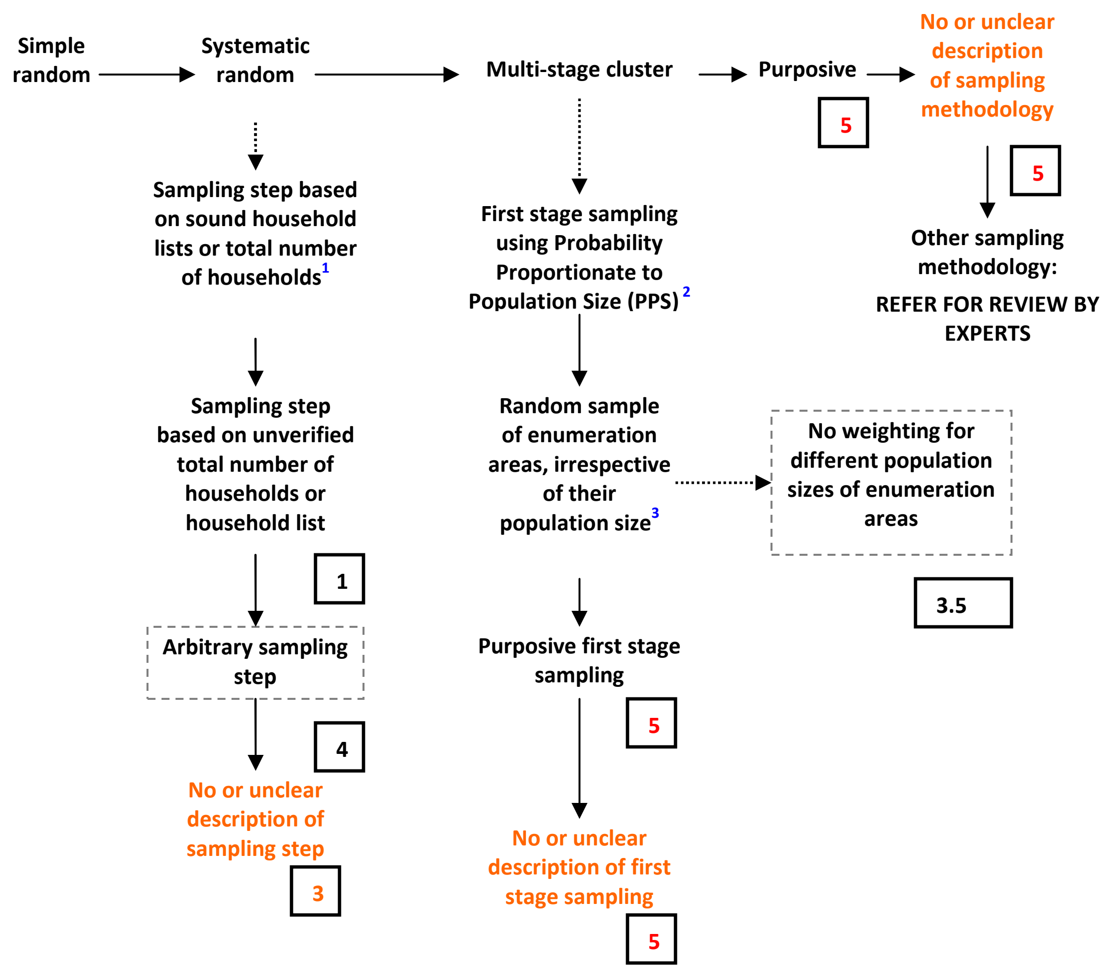

[1] If household lists or total numbers of households were not established by the survey investigators but were based on available sources of information, their veracity should have been verified.

[2] If use of SMART methodology is mentioned, it can be assumed that PPS is used.

[3] Including spatial sampling

1. SAMPLING - SELECTION BIAS (continued.)

1.3 Cluster sampling: Last stage household (HH) selection

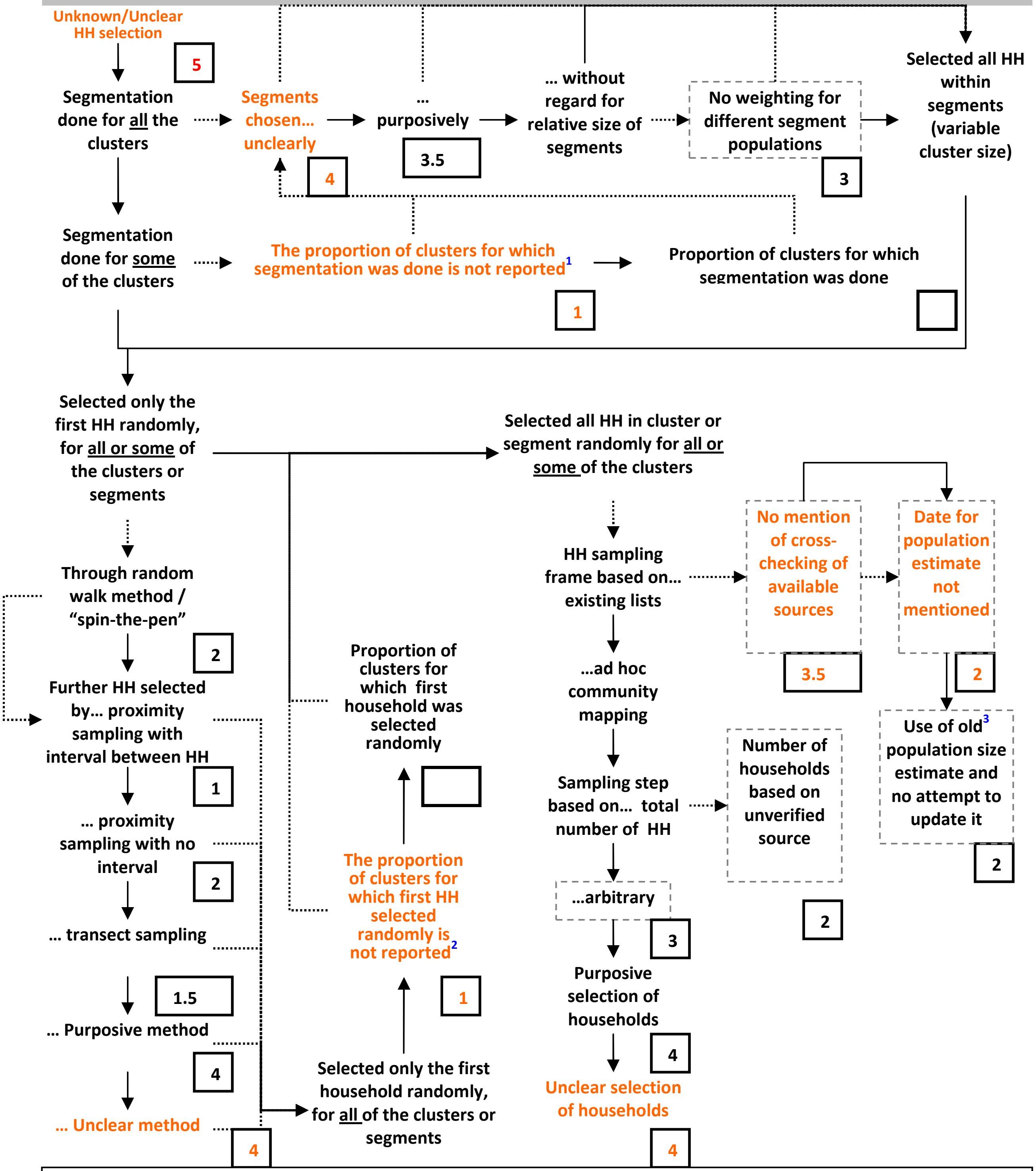

[1] Software automatically assigns a proportion of : 100 % if segmentation done for all clusters ; 0% if segmentation not done for any cluster; 50% if segmentation done for some clusters but proportion not reported

[2] Software automatically assigns a proportion of: 100 % if only first HH selected randomly for all clusters; 0% if this methodology not used for any cluster; 50% if this methodology used for some clusters but proportion not reported.

[3] Indicative benchmark, 3 months for unstable population, 3 years for stable population.

1. SAMPLING - SELECTION BIAS (continued)

1.4 Survey non-response [1]

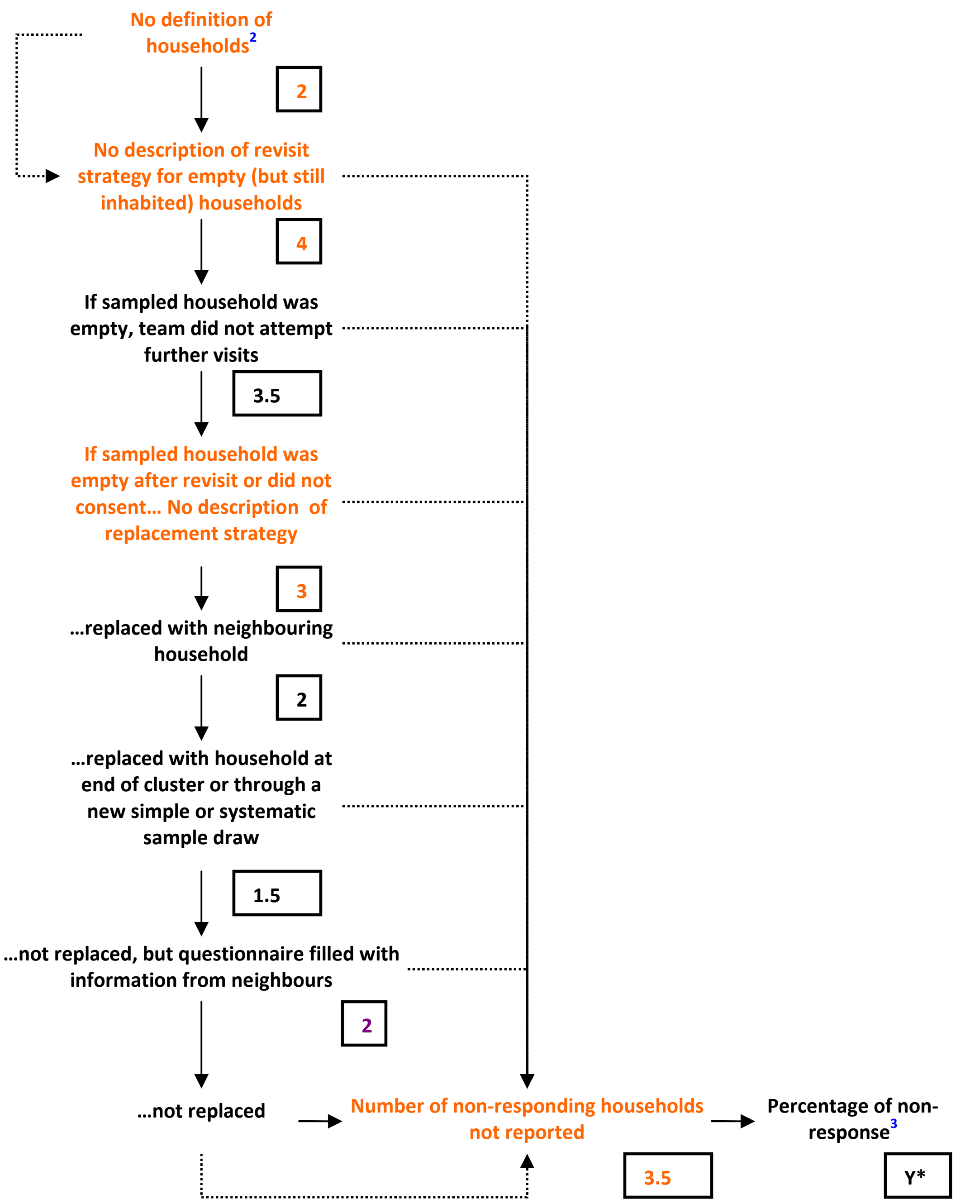

*points deducted by categories with an exponential relationship, i.e. <5% = 0, 5-9.9% = 1; 10-14.9% = 2; 15-24.9% = 3; >= 25% = 4

[1] Only descriptions for households should be considered, not for children; e.g. description of strategy for absent children should not be considered.
[2] A household should be clearly defined, such as "persons eating from the same cooking pot"
[3] If numbers are given in the survey report, % can be calculated

1. SAMPLING - SELECTION BIAS (continued)

1.5 Other selection biases mortality

Survey is nested within a nutritional survey, and

… only households with children eligible for anthropometry are included

4

… no evidence that households without children eligible for anthropometry are included

4

Any other mechanism is suspected whereby certain households or household members have an unequal probability of selection, other than due to stratification or any explicit sampling design characteristics considered above

REFER FOR REVIEW BY EXPERT SURVEY METHODOLOGIST, who should decide on any further points deduction, and/or on whether reanalysis is possible to rectify the problem

2. SELECTION - PRECISION Custer Sampling

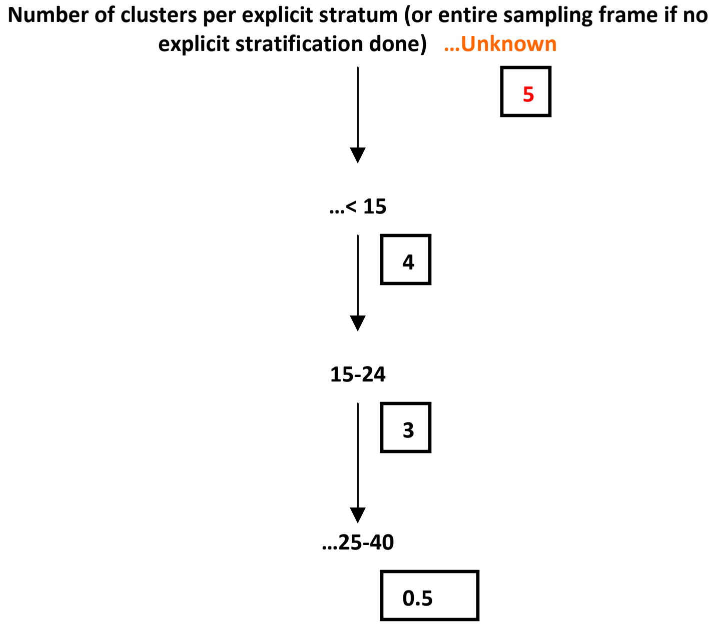

3. MEASUREMENT BIAS MORTALITY

3.1 Questionnaire

No evidence of the use of a structured questionnaire (not only tally sheet) including explicit questions and sequences of questions[1] → 4.5

No evidence that questionnaire was translated in local language and back translated, if population did not speak the same language as the investigator → 3

No evidence that questionnaire/tally-sheet pre-piloted before data collection[2] → 3

If multi-purpose survey, no evidence that mortality questions at the beginning of the interview and before anthropometric measurements, if relevant → 2

Aggregate (i.e. non-individual) questionnaire/tally-sheet only[3] → 2.5

Individual questionnaire/tally-sheet but current household census only[4] → 2

Past or hybrid household census, but no questions on deaths among visitors and newborns during period → 2

No or unclear description of type of census used → 2.5

No evidence that recall period has an easily recognisable starting date, e.g. religious event, first rainfall of the year → 4

No evidence of use of a calendar aid with salient dates for estimating dates of death[5] → 3.5

3.2 Training and supervision of data collection

No evidence that interviewers were trained before data collection → 3

No evidence of field supervision, including direct observation of interviews by investigators → 3

No evidence that data collection forms were checked at the end of each day by investigators → 2

[1] A structured questionnaire means that questions should be clearly written in order that exactly the same wording and sequence of questions is used with each family.

[2] Pre-piloting of mortality questionnaire and/or tally sheet should be explicitly mentioned.

[3] Aggregate vs. individual: the questionnaire/tally sheet should be considered aggregated if only information about the total number of persons in the family is asked/recorded, even if deaths are considered individually. Individual questionnaire/tally sheet should be considered when the members of the family are enumerated.

[4]Current household census: # of people living in the HH at the time of the survey (i.e. end of recall period) as opposed to past census (i.e. # of people living in the HH at the beginning of the recall period)

[5] Evidence should be considered only if the use of a calendar for estimating date of deaths is clearly stated (a local calendar might have been used for estimating age of children for nutrition, but not for dates of death).

4. MEASUREMENT BIAS NUTRITION

4.1 Child selection and measurements

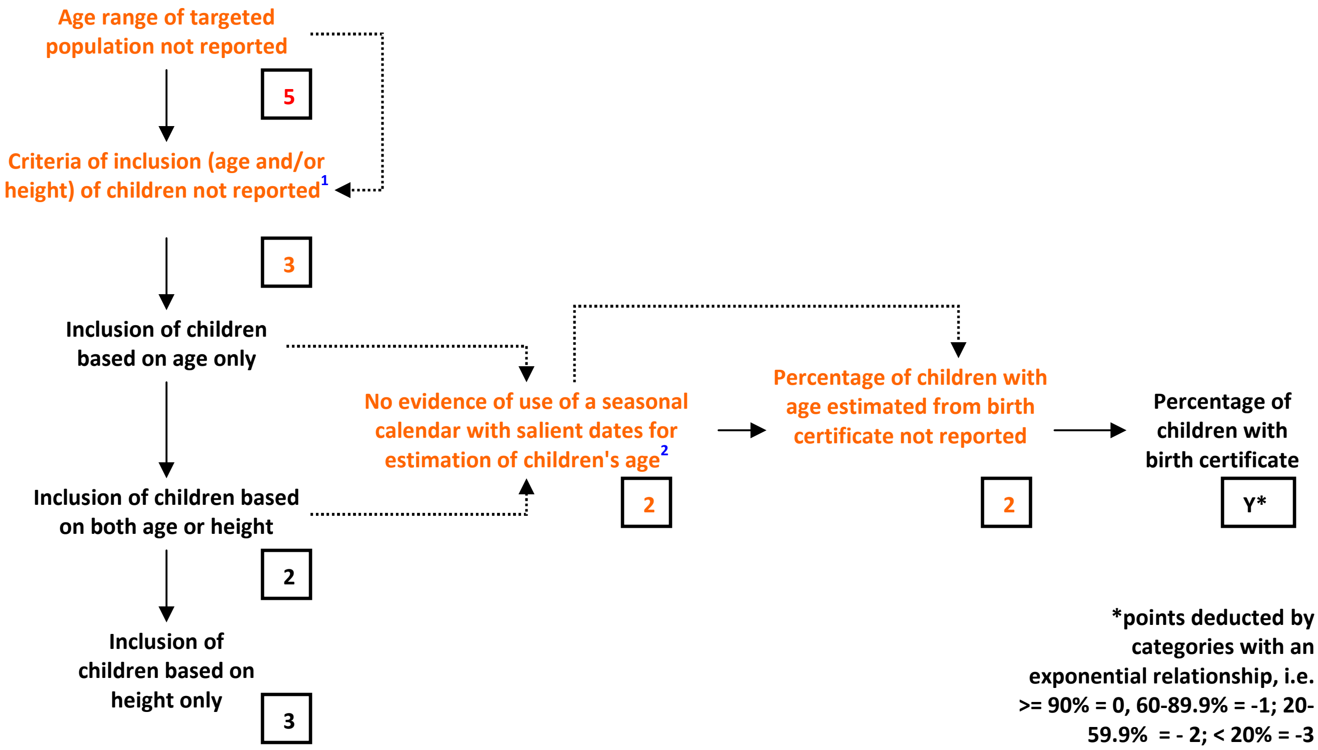

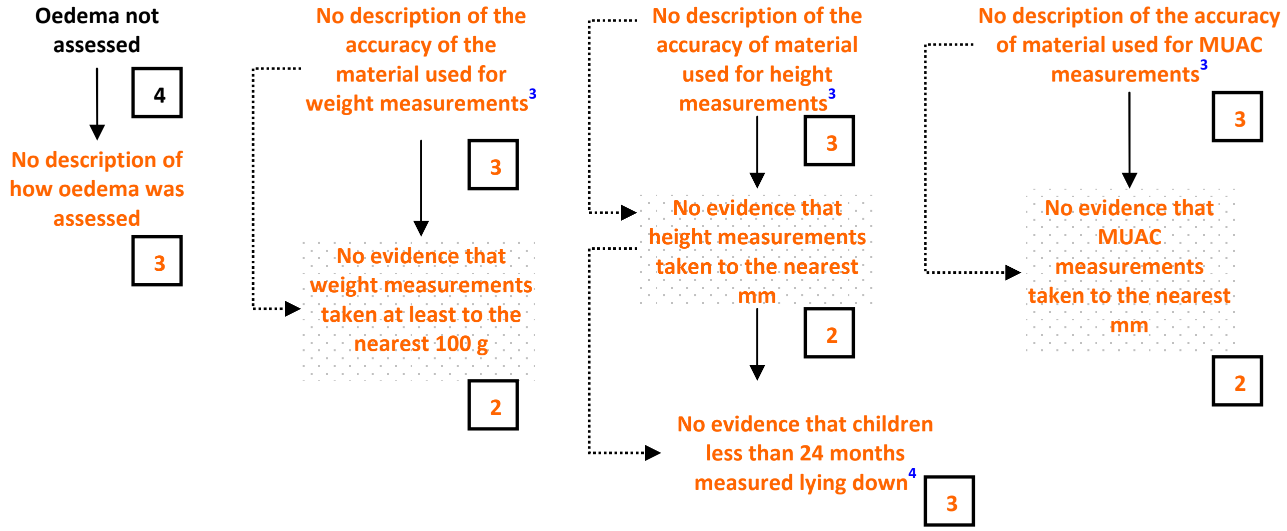

[1] Criteria of inclusion of children should be clearly mentioned. This is different from targeted population.

[2] Use of a seasonal calendar with salient date should be clearly mentioned or a calendar should be in the annex.

[3] Accuracy includes type of materials (e.g. bathroom scales are not accurate enough) , age of materials, and checking of accuracy against known weight or height.

[4] Height is often mentioned instead of age. This is OK.

4. MEASUREMENT BIAS NUTRITION (Continued)

4.2 Training and supervision of data collection

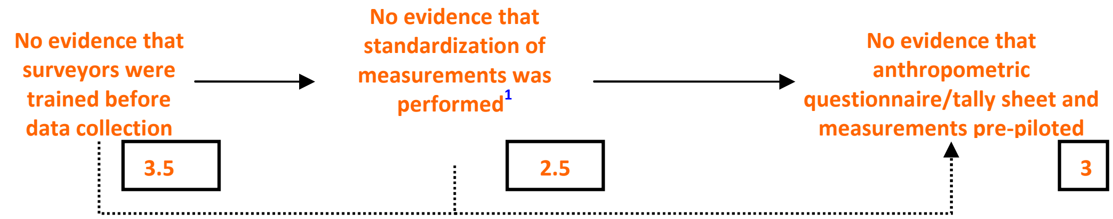

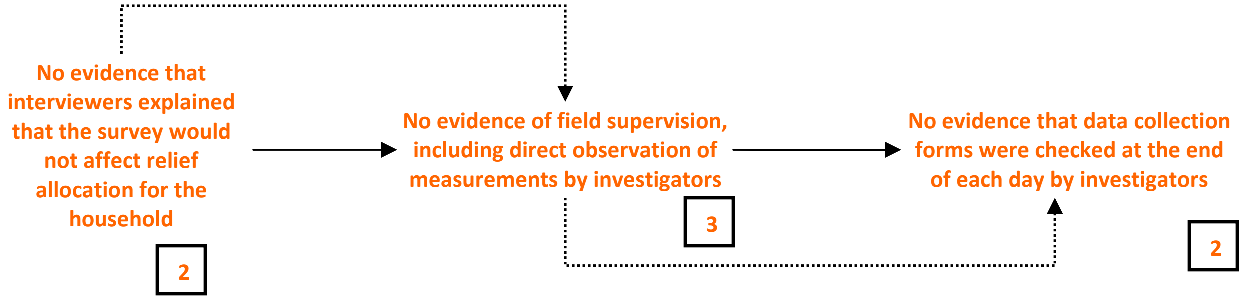

[1] Standardization of measurements refers to the methodology mentioned in SMART manual and WHO "Measuring change in nutritional status", i.e. 3 measurements of 10 children by measurers and supervisor and comparison of intra and inter-variability of measurements

5. RESPONSE BIAS MORTALITY

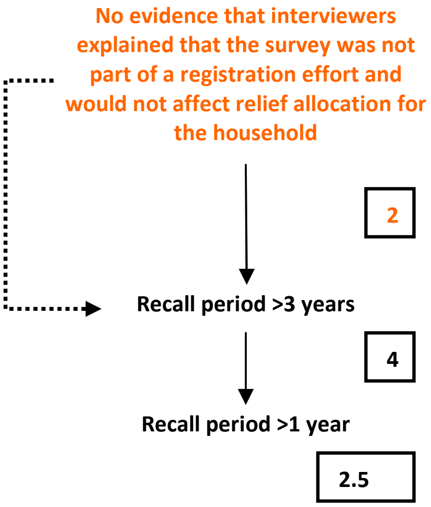

6. ANALYSIS MORTALITY

6.1 Stratification

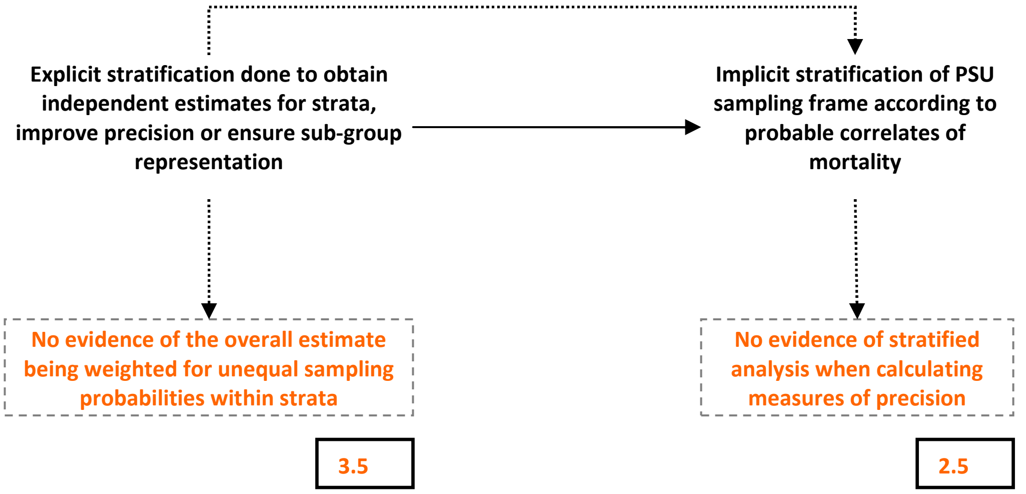

6.2 Calculation

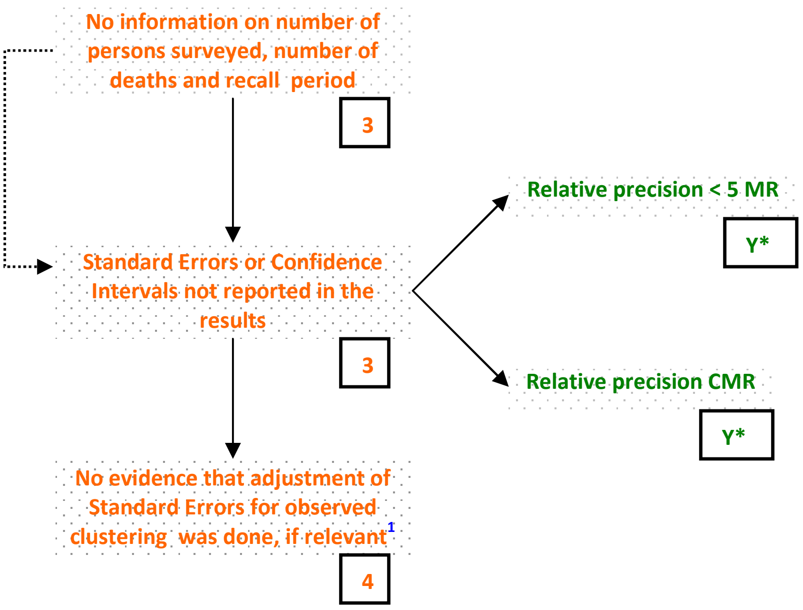

* Relative precision = [((point estimate - lower CI) + (upper CI - point estimate))/2]/ point estimate

Calculation done automatically by software; results shown in data.

Points deducted by categories with an exponential relationship, i.e. < 15% = 0; 15-34.9% = -1 ; 35-49.9% = - 2; 50-59.9% = -3; >= 60%= -4

[1] Either adjustment for clustering is specifically mentioned in the report, cluster design effect is given in the report, or there is mention of the use of software application such as ENA, ENA for Epi-Info and C-Sample module of EPI 6, which takes design effect into account when calculating Confidence Intervals of cluster surveys.

7. ANALYSIS NUTRITION

7.1 Stratification

Explicit stratification done to obtain independent estimates for strata, improve precision or ensure sub-group representation

No evidence of the overall estimate being weighted for unequal sampling probabilities within strata

3.5

Implicit stratification of PSU sampling frame according to probable correlates of mortality or nutrition

No evidence of stratified analysis when calculating measures of precision

2.5

7.2 Calculation

No information on number of children surveyed and number of children malnourished

3

No information on number of data discarded from the analysis because of inclusion errors (age/height) or anthropometric flags

2.5

Percentage of data discarded from the analysis

Y*

*points deducted by categories with an exponential relationship, i.e. <5% = 0, 5-9.9% = -1; 10-14.9% = - 2; 15-24.9% = -3; >= 25% = -4

Only one child measured per household and no evidence of weighting

2.5

Standard Errors or Confidence Intervals not specified in the results

3

Relative precision of SAM

Y*

Relative precision of GAM

Y*

* Relative precision = [((point estimate - lower CI) + (upper CI - point estimate))/2]/ point estimate
Calculation done automatically by software; results shown in data.

Points deducted by categories with an exponential relationship, i.e. < 15% = 0; 15-34.9% = -1 ; 35-49.9% = - 2; 50-59.9% = -3; > 60% = -4

No evidence that adjustment of Standard Errors for observed clustering was done, if relevant[1]

4

[1] Either adjustment for clustering is specifically mentioned in the report, cluster design effect is given in the report, or there is mention of the use of software application such as ENA, ENA for Epi-Info, EpiNut of Epi 5 and C-Sample module of EPI 6, which take design effect into account when calculating Confidence Intervals of cluster surveys.

7. ANALYSIS NUTRITION (Continued)

7. 2 Calculation (continued)

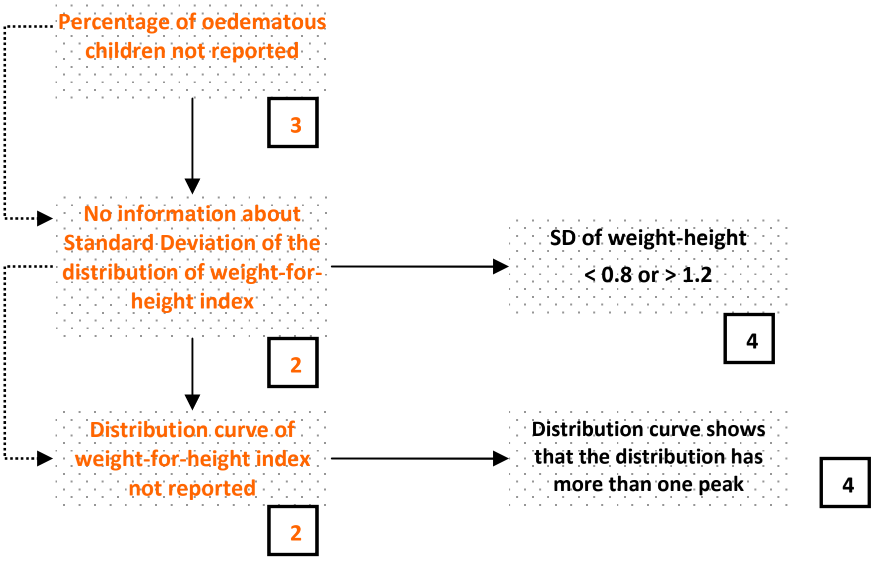

Supplement: Additional file 1 — shows the algorithm for checking the quality of mortality and nutrition surveys. [file 1478-7954-9-57-S1.PDF]
